# Informative predictors of pregnancy after first IVF cycle using eIVF practice highway electronic health records

**DOI:** 10.1038/s41598-022-04814-x

**Published:** 2022-01-17

**Authors:** Tingting Xu, Alexis de Figueiredo Veiga, Karissa C. Hammer, Ioannis Ch. Paschalidis, Shruthi Mahalingaiah

**Affiliations:** 1 Center for Information and Systems Engineering, Boston University, 8 St. Mary’s St, Boston, MA 02215 USA; 2 Department of Environmental Health, Harvard T.H. Chan School of Public Health, Building 1 655 Huntington Avenue, Building 1, 14th floor, Boston, MA 02115 USA; 3 Division of Reproductive Endocrinology and Infertility, Department of Obstetrics and Gynecology, Massachusetts General Hospital, 55 Fruit Street Yawkey 10, Boston, MA 02114 USA; 4 Division of Systems Engineering, Department of Electrical and Computer Engineering, Boston University, 8 St. Mary’s St, Boston, MA 02215 USA; 5 Department of Biomedical Engineering, Faculty of Computing and Data Sciences, Boston University, 8 St. Mary’s St, Boston, MA 02215 USA

**Keywords:** Infertility, Computational science

## Abstract

The aim of this study is to determine the most informative pre- and in-cycle variables for predicting success for a first autologous oocyte in-vitro fertilization (IVF) cycle. This is a retrospective study using 22,413 first autologous oocyte IVF cycles from 2001 to 2018. Models were developed to predict pregnancy following an IVF cycle with a fresh embryo transfer. The importance of each variable was determined by its coefficient in a logistic regression model and the prediction accuracy based on different variable sets was reported. The area under the receiver operating characteristic curve (AUC) on a validation patient cohort was the metric for prediction accuracy. Three factors were found to be of importance when predicting IVF success: age in three groups (38–40, 41–42, and above 42 years old), number of transferred embryos, and number of cryopreserved embryos. For predicting first-cycle IVF pregnancy using all available variables, the predictive model achieved an AUC of 68% + /− 0.01%. A parsimonious predictive model utilizing age (38–40, 41–42, and above 42 years old), number of transferred embryos, and number of cryopreserved embryos achieved an AUC of 65% + /− 0.01%. The proposed models accurately predict a single IVF cycle pregnancy outcome and identify important predictive variables associated with the outcome. These models are limited to predicting pregnancy immediately after the IVF cycle and not live birth. These models do not include indicators of multiple gestation and are not intended for clinical application.

## Introduction

According to the U.S. Centers for Disease Control and Prevention (CDC) report from the National Survey of Family Growth from 2015 to 2017, 13.1% of women aged 15–49 in the U.S. have impaired fecundity and 12.7% of women aged 15–49 have used some type of infertility services^1^. The process of infertility assessment, management, and in vitro fertilization (IVF) treatment can be time-consuming, emotionally draining and financially burdensome, even in mandated states. IVF success rates by clinic have been compiled by the Society for Assisted Reproductive Technology (SART), which also provides aggregated national success rates that may not sufficiently adapt to individual subject characteristics^2^.

Currently two prediction models are widely used to predict IVF success rate by individual characteristics: the SART Patient Predictor, and Univfy PreIVF Report. The SART Patient Predictor, a free resource, uses demographic information on a patient’s age, weight, height, infertility diagnosis, and previous pregnancy outcomes to predict chances of live birth after one to three IVF cycles^3^. Alternatively, for a cost, the Univfy PreIVF Report uses an individual’s age, body mass index (BMI), ovarian reserve test results, reproductive history and clinical diagnosis to predict their pre-IVF success chances^4^. Each of these tools is useful for the individual that is considering undergoing IVF. However, in cycle information such as peak estradiol levels prior to retrieval or the number of oocytes retrieved was not considered in these models. Such information may add to the accuracy of outcome prediction when patients undergo embryo transfer which could help prepare them psychologically for a positive or negative pregnancy outcome within the first treatment cycle.

Therefore, the likelihood of success estimated prior to and during the first IVF cycle offers important information for both the patient and their physician. This study seeks to elucidate the most important prediction variables such as pre-cycle demographic characteristics and in-cycle factors, that lead to pregnancy after the first IVF treatment cycle with fresh embryo transfer. An additional objective is to establish a highly accurate predictive model that is simple, interpretable, and easy to compute.

## Materials and methods

### Data acquisition

This study collected de-identified patient data from 2001 to 2018 from clinics that use eIVF practice highway for their electronic medical records. Participating practices included multiple IVF centers in Massachusetts, New York and California. Inclusion criteria were as follows: women undergoing their first IVF cycle in eIVF using autologous oocytes and planning at least one embryo transfer. Exclusion criteria included: use of donor oocytes, those with no embryo transfers, and any subsequent IVF cycles. The study was determined to be non-human subjects research and approved by the pertinent institutional review board (IRB) of the Boston University Medical Campus (BUMC) (H-37693). Our study methods and analysis conform to the guidelines and regulations set by the H-37693 agreement with the BUMC IRB. Given that our study was approved as a non-human subjects research, the need to obtain informed consent was waived by the BUMC IRB.

Success of the first IVF cycle was identified as pregnancy after embryo transfer, which was confirmed by having any of the following three: a positive serologic bHCG, ultrasound with intrauterine pregnancy, or a live birth. As for the predictors obtained from the eIVF records, we considered the following demographic and lifestyle pre-cycle variables: age (< 35, 35–37, 38–40, 41–42, 42+), race (Asian, Black/African American, Hispanic/Latina, White/Caucasian, unknown), body mass index (BMI) in kg/m^2^ (< 18.5, 18.5–24.9, 25.5–29.9, 30.0–34.9, 35–39.9, 40+), alcohol use (yes/no), tobacco use (yes/no), exercise (days/week). Pre-cycle fertility evaluation variables included: anti-mullerian hormone (AMH), antral follicle count (AFC), estradiol levels, follicle stimulating hormone (FSH) levels, luteinizing hormone (LH) levels, endometrial thickness (mm), and male partner semen analysis parameters including specimen volume, sperm concentration, sperm motility, and sperm progression. Medical diagnoses were categorized in the following manner: female infertility, male infertility, unexplained infertility, tubal disease, endometriosis, and ovulatory dysfunction (including ovulation problems, polycystic ovary syndrome (PCOS), and ovarian failure/diminished reserve). In-cycle variables included were: maximum estradiol value, number of oocytes retrieved, number of transferred embryos, and number of embryos cryopreserved.

Assuming that all individuals’ first cycles were independent, predictive models were built on a positive pregnancy outcome (i.e., positive pregnancy test/clinical pregnancy/live birth) determination after embryo transfer using logistic regression, support vector machines, random forest and gradient-boosted decision trees.

### Data pre-processing

Records of IVF subjects undergoing their first autologous oocytes IVF cycle within the de-identified eIVF practice highway dataset were extracted. All categorical variables were encoded to generate numerical input values for the machine learning models. Each outcome from a categorical variable was represented by a new variable representing the occurrence of this value (binary variable). For instance, the “age” variable takes five possible values {< 35, 35–37, 38–40, 41–42, 42 +}, so five distinct new indicator variables were generated: {age < 35, age 35–37, age 38–40, age 41–42, age 42+}. In a similar fashion, binary variables were created for race categories {Asian, Black/African American, Hispanic/Latina, White/Caucasian, unknown}. The created variables are binary and can only take values from {1, 0}.

For the initial analysis, those variables with more than 99% missing data were excluded. Data imputation was also conducted to address missing values. For continuous variables, the missing values were replaced with the median of non-missing values. For categorical variables, an indicator variable was used to represent the occurrence of the missing values. For each variable, outliers that were greater than the 99th percentile or less than the 1st percentile were replaced with the 99th percentile or the 1st percentile. respectively. To prevent collinearity, for pairs of highly correlated variables (with absolute value of correlation coefficient > 0.8), only one of them was retained. A linear transformation was conducted to the data before training predictive models. The z-score transformation^5^ was adopted to linearly transform the variables to have mean 0 and standard deviation 1.

### Predictive models

Multiple models were trained to predict IVF success in our data, including linear models such as regularized logistic regression using L1- and L2-norm penalties (L1LR and L2LR, respectively), L1-and L2-regularized support vector machines (L1SVM and L2SVM, respectively), and nonlinear models such as random forests and gradient-boosted decision trees. Modeling and data analysis was conducted using Python ﻿3.7.0.

In the classic logistic regression model, the probability of the outcome can be expressed as a logit function of a linear combination of input variables, with weights of the input variables as parameters. In order to increase the interpretability of the classifier, L1- and L2-norm regularization was used; specifically, an extra penalty term proportional to the L1-norm (L2-norm) of the parameters was introduced. The L1-regularized model is known to select a sparse subset of informative variables^6,7^.

The support vector machine (SVM)^8–10^ is another binary classification algorithm, which computes a high-dimensional plane with maximal margin to separate samples from different classes. Similar to logistic regression model, L1-regularized and L2-regularized SVM were used.

Random forest (RF)^11–13^ is an ensemble algorithm which builds multiple base decision tree models in parallel. Each tree is a model with different combination of covariates and computes predictions (‘votes’). The algorithm then adopts the majority vote of all base decision tree model predictions as the final classification results. RF uses the bootstrap-aggregating algorithm, also called bagging, to randomly generate subsets of samples and variables from the original data set to train the base decision tree, which reduces the correlation between different decision trees and thus effectively improves the stability of the ensemble algorithm and prediction accuracy.

Extreme gradient boosting (XGBoost)^12,14–16^ is also an ensemble of multiple decision tree models; but it uses a large number of shallow trees (weak learners) built in a sequential manner. Each of these trees is trained to correct the mistakes in the previous model in the series. With systems optimization techniques, such as parallelization, cache optimization, distributed computing, XGBoost is able to scale beyond billions of samples with much fewer resources compared to existing systems.

### Experimental settings

Predictive models as described above were created to predict whether subjects would become pregnant after the first autologous IVF treatment cycle with fresh embryo transfer. The input data was divided into a training set (80%) and a test set (20%). The predictive models were trained only using the subjects’ variables and labels in the training set. The prediction performance of the trained models was evaluated on the test set. To obtain the best predicting performance of all algorithms, five-fold cross-validation over the training set was conducted to select the optimal hyper-parameters, for these algorithms and models, such as the regularization parameters in L1LR, L2LR, L1SVM, L2SVM, and the number and maximum depth of the trees in RF and XGBoost. All the classification models were trained and evaluated ten times with different randomly generated training/test sets and the average and standard deviation of the performance parameters over these ten runs were reported.

To evaluate model performance the area under the curve (AUC) of the receiver operating characteristic (ROC) curve on the test set was reported. The ROC plots the sensitivity of the model against one minus the specificity as one varies the classification threshold for deciding positive vs. negative outcomes.

The best performing L2LR model was utilized to examine the corresponding coefficients. Variables in the predictive model were ranked by the absolute values of the corresponding model coefficients. Variables with a coefficient *p*-value < 0.05 in the hypothesis test of being zero and with an absolute coefficient no less than ten percent of the absolute value of the largest absolute coefficient were tabulated. The reference groups for age, race and BMI were ‘age < 35’, ‘race unknown’, and ‘BMI category 18.5–24.9’, respectively. The performance and model parameters of parsimonious models using only the top five most important variables were also reported. The z-score normalized variables were denoted by ${\text{x}}_{1} ,{\text{ x}}_{2} ,{\text{ x}}_{3} ,{\text{ x}}_{4} ,{\text{ x}}_{5}$, respectively. A simple formula from the best L2LR model to calculate the probability of a subject to be classified as pregnant is ${\text{p }} = { }\frac{{e^{f} }}{{1 + e^{f} }}$, where ${\text{f }} = { }0.35x_{1} + 0.13x_{2} - 0.31x_{3} - 0.24x_{4} - 0.16x_{5} + 0.07$.

### Secondary analysis

As a secondary analysis, we evaluated first IVF cycle with fresh embryo transfer success rate by race, physical activity, and alcohol consumption in our study subjects. We computed and reported the odds ratio (OR) and 95% confidence interval (95% CI) for these variables from the L2LR model with all variables included (Supplementary Tables S3 and S4).

## Results

### Results of data acquisition and data pre-processing

The initial data pull resulted in 41,771 subjects from which 22,413 IVF cycles satisfied the selection criteria (first autologous oocyte IVF cycle with planned fresh embryo transfer). The majority of participants (88.3%) were 40 years old or younger, 20% identified as White/Caucasian and 4% as Asian. Of note, there were 73.3% missing responses for the race variable. The BMI of 38.9% of participants fell between 18.5 and 24.9 kg/m^2^, which is considered normal, and 20.3% were found to be overweight with BMI between 25.5 and 29.9 kg/m^2^. The overall pregnancy rate after the first cycle with fresh embryo transfer within the cohort was 52% (n = 11,584). The overall distribution of the subjects’ demographic characteristics and diagnoses is listed in Table 1. Supplementary Table S1 describes the variables used by the predictive models. There were 24 binary variables and 14 continous variables retained as predictors after the data pre-processing procedures.

**Table 1:** Demographic characteristics of patients undergoing in vitro fertilization (IVF) treatment included in our study.

Category	All 22,413 subjects
**Age categorized**	
< 35	10,698 (47.7%)
35–37	5011 (22.4%)
38–40	4078 (18.2%)
41–42	1738 (7.8%)
42+	888 (4.0%)
**Race/ethnicity categorized**	
White/Caucasian	4484 (20%)
Asian	898 (4.0%)
Hispanic/Latina	397 (1.8%)
Black/African American	205 (0.9%)
Unknown	15,266 (73.3%)
**BMI**	
< 18.5	452 (2%)
18.5 -24.9	8709 (38.9%)
25.5 -29.9	4547 (20.3%)
30.0 -34.9	1935 (8.6%)
35.0 -39.9	1044 (4.7%)
40 +	776 (3.5%)
**Diagnosis**	
Female infertility	5295 (23.6%)
Unexplained infertility	3402 (15.2%)
Ovulatory dysfunction	2259 (10.1%)
Endometriosis	902 (4%)
Tubal disease	896 (4%)
Male infertility	432 (1.9%)

### Results of predicting pregnancy after first IVF cycle

Table 2 shows the mean and standard deviation of AUC from all classification methods over 10 runs for predicting pregnancy after the first IVF cycle. All variables that are left after data preprocessing were used. XGBoost achieves the best performance among all algorithms with an average AUC of 0.68. Table 2 also shows the most important variables in the predictive model, ranked by the absolute values of the corresponding model coefficients.

**Table 2:** IVF pregnancy prediction model: performance metrics and most significant variables.

Performance for IVF pregnancy prediction							
Algorithm	Mean AUC	Std AUC	Algorithm	Mean AUC	Std AUC		
L1LR	0.6630	0.0084	L1SVM	0.6630	0.0090		
L2LR	0.6632	0.0091	L2SVM	0.6630	0.0091		
XGBoost	0.6783	0.0097	RF	0.6750	0.0087		

Most significant variables for IVF pregnancy prediction ranked by absolute value							
Rank	Variables	Coef	Coef 95% CI	Y1 mean	Y0 mean	*p*-value	Y-corr
1	Age 42 +	−0.314	[−0.348, −0.279]	0.02	0.07	< 0.001	−0.13
2	Number of cryopreserved embryos	0.256	[0.22, 0.293]	2.33	1.29	< 0.001	0.19
3	Age 41–42	−0.248	[−0.28, −0.217]	0.05	0.11	< 0.001	−0.10
4	Age 38–40	−0.172	[−0.202, −0.141]	0.16	0.21	< 0.001	−0.06
5	Count of Transferred Embryos	0.156	[0.125, 0.186]	1.89	1.94	< 0.001	−0.03
6	Max Estradiol	0.100	[0.067, 0.133]	2430.70	2067.66	< 0.001	0.14
7	Diagnosis of Unexplained Infertility	0.090	[0.062, 0.119]	0.17	0.14	< 0.001	0.05
8	Race White/Caucasian	0.089	[0.06, 0.117]	0.22	0.18	< 0.001	0.06
9	Age 35–37	−0.077	[−0.106, −0.048]	0.23	0.22	4.79E−01	0.01
10	Currently Exercise	0.072	[0.041, 0.103]	0.31	0.25	< 0.001	0.07
11	Diagnosis of Tubal Disease	−0.071	[−0.099, −0.043]	0.04	0.04	6.88E−03	−0.02
12	Number of Retrieved Oocytes	0.057	[0.019, 0.094]	12.74	10.37	< 0.001	0.16
13	Sperm Concentration	0.047	[0.017, 0.077]	28.53	27.04	< 0.001	0.03
14	Sperm Motility	−0.041	[−0.068, −0.013]	68.43	69.66	< 0.001	−0.02
15	Sperm Volume	0.038	[0.008, 0.068]	1.27	1.17	< 0.001	0.04
16	Race Black/African American	−0.036	[−0.064, −0.009]	0.01	0.01	1.32E−02	−0.02

IVF pregnancy prediction model: performance metrics and most significant variables. We list the LR coefficients of statistically significant variables (Coef) and their 95% confidence intervals, the correlation of the variable with the pregnancy label (Y-corr), the mean value of the variable (Y1-mean) in the pregnant class, and the mean value of the variable (Y0-mean) in the non-pregnant class. *p*-values were computed to compare the mean of each variable in the two cohorts (pregnant and non-pregnant) using a two-sided t-test, with the null hypothesis being that the two means are equal.

The five most significant predictive variables include two in-cycle variables: ‘number of transferred embryos’ (model coefficient = 0.16) and ‘number of cryopreserved embryos’ (model coefficient = 0.26), and three demographic variables referring to age: ‘age 38–40’ (model coefficient = -0.17), ‘age 41–42’ (model coefficient = −0.25), and ‘age 42 + ’ (model coefficient = −0.31). With only the five most informative predictive variables, the L2LR model provided high prediction accuracy with mean AUC of 0.65 and standard deviation of 0.0088 over 10 random runs. The corresponding model coefficients and 95% confidence intervals are listed in Table 3.

**Table 3:** Predictive IVF success model with only the 5 most important variables.

Most significant variables for IVF pregnancy prediction							
	Variables	Coef	Coef 95% CI	Y1 mean	Y0 mean	*p*-value	Y-corr
1	Number of cryopreserved embryos	0.35	[0.32, 0.38]	2.33	1.29	< 0.001	0.19
2	Age 42+	−0.31	[−0.34, −0.27]	0.02	0.07	< 0.001	−0.13
3	Age 41–42	−0.24	[−0.27, −0.21]	0.05	0.11	< 0.001	−0.10
4	Age 38–40	−0.16	[−0.19, −0.13]	0.16	0.21	< 0.001	−0.06
5	Count of Transferred Embryos	0.13	[0.1, 0.16]	1.89	1.94	< 0.001	−0.03
	Model Intercept	0.07	[0.04, 0.1]				

### Results of the secondary analysis

After including all pre-processed variables, we found that White/Caucasian subjects had higher success in their first IVF cycle with fresh embryo transfer (OR 1.09, 95% CI 1.06–1.12) and Black/African American had lower success (OR 0.97, 95% CI 0.94–0.99), compared to those whose race was unknown as presented in Supplementary Table S3. Odds ratio for those who self identified as Asian and Hispanic/Latina were 0.98 (95% CI 0.96–1.01) and 1.00 (95% CI 0.98–1.03), respectively, with *p*-values > 0.05. Physical activity was ranked 10th in importance as a preditor for IVF and subjects who exercised had higher IVF success compared to those who did not exercise (OR 1.08, 95% CI 1.04–1.11). Alcohol consumption ranked 20th most important predictor of IVF success, and there was no odds association with IVF success when comparing those who did drink versus those who did not (OR 1.00, 95% CI 0.98–1.04) as seen in Supplementary Table S4.

## Discussion

We designed predictive models using pre-cycle and in-cycle variables to predict pregnancy after the first IVF cycle for those with at least one embryo transfer, using a large IVF electronic record-based dataset with 22,413 subjects. As far as we know, this is the first study to develop a predictive model that incorporates in-cycle parameters to gauge success rates for the ongoing IVF cycle. This may be a potentially useful tool in the future for patients to assess their real-time chance of conception after embryo transfer with the use of the in-cycle variables of number of embryos transferred and number of embryos cryopreserved among those who undergo a first fresh embryo transfer.

As expected, we found age to be an important predictor for pregnancy after the first IVF cycle. Three groups of the age variable were identified to have the greatest impact on the prediction of pregnancy: 38–40, 41–42, and above 42 years old when compared to those who were 35–37 years old. As is well known, advanced maternal age is associated with a lower chance of pregnancy^17–19^. Other variables such as race/ethnicity, BMI, and fertility diagnosis did not significantly improve prediction of IVF success for the first IVF cycle. Among the five most informative predictors of IVF success were two in-cycle variables: the number of embryos transferred and number of embryos cryopreserved. Additionally, in-cycle variables such as peak estradiol and number of oocytes retrieved were ranked as the 6th and 12th in importance as predictors, respectively. While these were not included in the parsimonious model (which used only the top 5 informative variables), they were included in the predictive model that used all available variables. Prasad, Sudha et al., also found that estradiol on day 2 and day of trigger was a successful predictor of pregnancy after IVF treatment with embryo transfer^20^. Pre-cycle factors, such cause of infertility (i.e., unexplained infertility and tubal disease) were also found to be informative predictors of IVF pregnancy as in previous studies^21,22^. Male-related factors such as sperm concentration, motility and volume were ranked 13th, 14th and 15th, respectively as predictors of pregnancy after first IVF cycle in our model. The literature has found sperm parameters to be insignificant predictors of IVF outcomes^21^.

Previous studies have investigated how the IVF success rate can be affected by either one or a combination of variables, such as age, AMH, oocyte yield and FSH using other aproaches in either bivariate or multivariable logistic regression^23–27^. Some prior studies, which assessed individual or combined predictive value of AMH, FSH, ovarian reserve tests, antral follicle count, age, inhibin B, number of oocytes retrieved, and serum LH concentration on stimulation day 1 provided limited prediction accuracy (AUC less than 0.6)^28–30^; whereas ours yielded an AUC close to 0.70. One possible reason for such improvement is that our models were developed based on a multicenter electronic medical record system data with a much larger sample size (> 20,000 IVF records) than most other studies^30–32^. Moreover, most of the predictive models in the literature are based on very complex algorithms such as ranking algorithms, random forest, Bayesian networks, and neural networks, which are difficult to interpret or apply to medical practice^31–34^. This study overcomes these challenges by demonstrating that limiting our model to the top five most important predictor variables still maintained a high accuracy, yielding an average AUC of 0.65 vs. an AUC of 0.68 when additional variables were used.

The pre-existing predictive models with high accuracy utilize pre-cycle variables only, making our model distinct in that it incorporates a number of pre-cycle and in-cycle variables. Our model is close in accuracy to that described by McLernon et al.; their prediction model for cumulative live birth from IVF has an AUC of 0.72 (0.71–0.73) and included pre-cycle variables such as height, weight, pregnancy history and age^35^. This prediction model is used to predict pregnancy success after multiple IVF cycles, whereas our goal is prediction from the first cycle attempt. Predicting IVF success after first cycle attempt, can be helpful for counseling couples in states without mandated care because they may only be able to undergo one IVF cycle treatment due to the cost prohibitive nature of this process. Another popular predictive model is the Univfy algorithm which predicts live birth for patients prior to starting an IVF cycle^36^. The Unifvy algorithm uses pre-cycle information about the patient but also includes male partner information. It can be used by patients or physicians that pay for the service; the variables used within the algorithm are not entirely publicly available^4^.

Although race was not identified as the most important (i.e., within the top 5) predictor of first IVF cycle and no solid conclusions can be drawn from analyzing this variable given the high missingness of 73.3%, we found White/Caucasian subjects had slightly higher odds of achieving pregnancy after the first IVF treatment than the unknown category while Black/African American subjects had slightly lower odds. For the Asian race category the odds of IVF success were slightly lower as well, but estimates were imprecise. Furthermore, no association between the Hispanic/Latina race category and IVF success was found in this analysis. The poor ascertainment of race is very common for large IVF datasets, including the SART registry. Under-reporting of the race variable, particularly in the SART database may have influenced race not being included in the publicly available predictor. In our study, while race may have been under reported in the eIVF electronic medical record sourced data, it is likely under-reported similarly across all race categories.

The association between race/ethnicity and IVF outcome has been limited in prior studies due to limitations in ascertainment of this variable. The few studies that have reported on this, have conflicting results with one study reporting no significant difference in IVF success between African American and White patients while another found that Caucasian ethnicity was an important independent predictor of clinical pregnancy^37,38^. Although race is not an inherent biological trait, it is a social construct that affects equalities in healthcare because of structural racism. The inclusion of race ascertainment in an IVF seeking population might enlighten racial/ethnic discriminations that negatively impact the health outcomes of patients seeking to become pregnant. As pointed out by Nancy Krieger, “although data by themselves cannot rectify health inequities, the absence of data demonstrating harm nevertheless is itself harmful”^39^. Furthermore, as researchers we must strive to “build public clarity about the extent and health consequences of racial discrimination” in order to advance health equity for all^39^.

Other epidemiological factors such as alcohol consumption have been shown to negatively impact IVF success in other studies^40^ but did not emerge in our model as an important predictor. In the secondary analysis, the alcohol consumption variable was ranked 20^th^ in importance as a predictor and the odds ratio estimate showed no association with IVF success. Physical activity, however, provided a slight improvement in odds of achieving IVF success in our cohort as seen in other studies^41^.

There are some important limitations to consider with this dataset. We did not include multiple embryo transfer attempts from the initial IVF cycle that yielded any cryopreserved gametes or embryos because the dataset did not readily identify such cycles. This dataset is limited to predicting IVF success among first cycle patients with at least one fresh transfer. To reach embryo transfer, a patient must undergo several successful processes including fertilization and subsequent embryo development. Therefore, a potential limitation to consider is that having at least one embryo transfer is considered as the starting point of first IVF cycle treatment in this analysis. Similarly, restricting analysis to those who had at least one embryo transfer in their first IVF cycle treatment can lead to exclusion of those whose first cycle treatment yielded cryopreservation for all embryos for genetic testing or medical purposes or if there were no emrbyos created from the cycle for other reasons such as poor oocyte yield or quality, or failed fertilization. Furthermore, due to differences in practice patterns and usage of the eIVF platform, diagnoses were reported in a heterogenous manner. We had to aggregate these diagnoses into broader categories, which might have led to minor misclassification of this variable set. An additional potential limitation is that the outcome of interest we predicted was pregnancy after the cycle but not live birth. The main reason for this was the heterogeneity of reported pregnancy outcomes, as some clinics reported only live birth while others reported ultrasound evidence or serologic evidence of pregnancy after IVF. Due to the variety of reporting we included all available outcomes (i.e., positive pregnancy test, ultrasound evidence of pregnancy and live birth) as a positive pregnancy result. In a future study including live birth as the main outcome measure, evaluation of other factors that may impact chance of live birth, such as risk factors for pregnancy loss including high BMI may be important predictors of IVF success. We were also unable to analyze time trends related to IVF practices with this dataset as we did not have access to dates in this de-identified dataset.

While not a true limitation but a consideration, the large dataset examined in this study was provided by the eIVF practice highway and there were two critical factors involved with its creation that may have affected data quality: (1) the availability of the variables requested by the research team in their clinics, and (2) the number of transactions taking place in the servers of each clinic at the time of the data pull. The data pull was balanced against the transaction burden of the eIVF interface at the level of the clinical site. Centers that had heavy transactions were excluded as to not slowdown their day-to-day activities. Finally, there was a limitation on the number of variables allowable at our initial data pull.

This study has several strengths. The use of a multicenter dataset makes our prediction models more widely applicable as they were formed utilizing data from three different states with three different mandates for IVF insurance coverage. Studies have demonstrated that state mandates lead to different assisted reproductive technology (ART) utilization rates, e.g., Sunderam et al. reported an ART utilization rate in Massachusetts of 1.5 times that of non-mandated states^42^. Provost et al. described differences in embryo transfer practices, resulting in lower multiple birth rates in mandated states compared to non-mandated states^43^. As noted in the study, number of embryos transferred was predictive of pregnancy. Number of embryos transferred should be balanced with the risk involved with multiple pregnancy and this is a challenging consideration if couples have a financial burden of cost of IVF cycle which might influence them to request multiple embryo transfer. Caution should be made when using this model in clinical counseling as this study was designed to use machine learning techniques in electronic health records but it does not substitute American Society for Reproductive Medicine guidance to optimize pregnancy and health of pregnancy and maternal/child health^44^. Given the impact state mandates can have on management of ART, it is important that we included states with different insurance coverage statuses. At the time of this study, Massachusetts requires all insurers that provide pregnancy-related benefits to also provide coverage for diagnosis and treatment of infertility, including IVF procedures. New York mandates insurance companies to cover diagnosis of infertility and medications for treatment but not IVF procedures. California requires insurance companies to offer coverage for fertility treatments excluding IVF^45^.

The advantage of this work is that it not only utilizes a large dataset to provide a high-accuracy IVF success prediction model, but also quantitatively measures the impact of multiple variables on the cycle outcome. The proposed simple classification model includes a pregnancy probability calculation formula that includes both pre-cycle and in-cycle variables to predict success of the first IVF cycle with autologous oocytes. The predictive models can be easily adapted to other disease predictions (e.g., poor ovarian response) with more variables. Application of this model can be specialized or generalized based on availability of well ascertained IVF datasets.

## Conclusion

In conclusion, an average AUC of 0.68 was achieved in the proposed IVF first cycle pregnancy prediction model. Pre-cycle variables—age, and in-cycle variables—number of cryopreserved embryos and number of transferred embryos were among the most informative variables. Even when restricted to the five most informative variables, the IVF pregnancy predictive model achieved an AUC of 0.65. This model should be applied taking into consideration its limitations such as amount of missingness with the race variable and study design (i.e. pregnancy was the outcome measured instead of live birth). Although poorly ascertained in this dataset, race was found to be of certain importance in predicting IVF success and further research should further evaluate its role in prediction of IVF outcomes. Additional research will be needed to develop predictive models for those undergoing subsequent cycles using cryopreserved embryos, using pre-implantation genetically tested (PGT) embryos, or including multiple cycles per woman.

## Supplementary Information

Supplementary Information.

## Data Availability

Data is available upon reasonable request to the corresponding author.
