# Supplementary material for: Informative predictors of pregnancy after first IVF cycle using eIVF practice highway electronic health records
Source: Sci Rep. 2022 Jan 17;12:839. doi: 10.1038/s41598-022-04814-x (PMC8763861; doi:10.1038/s41598-022-04814-x)
Supplement: Supplementary file 1 — Supplementary Information. [file 41598_2022_4814_MOESM1_ESM.pdf]

Supplementary Table S1. Description of Various Types of Medical Variables in the IVF Data.

Ontology	Examples
**Demographics**	Age (years)<br>Race (White/Caucasian, Asian, Hispanic/Latina, Black/African American, unknown)
**Vital Signs**	Body Mass Index (BMI - defined as kg/m^2)
**Oocyte & Embryo**	Oocytes retrieved, number of cryopreserved embryos, number of transferred embryos
**Sperm**	Sperm volume, concentration, motility, and progression
**Lifestyle**	Smoke (current, yes/no), alcohol use (current, yes/no), exercise (current, yes/no)
**Serologic Hormone Values**	Day 3 ovarian test values: estradiol (E2), luteinizing hormone (LH), follicle stimulating hormone (FSH), endometrial thickness, antral follicle count (AFC), max estradiol, last anti mullarian hormone (AMH) value
**Diagnoses Groups**	Female infertility, male infertility, unexplained infertility, tubal factor infertility, endometriosis, and ovulatory dysfunction

Supplementary Table S2. Performance of IVF outcome prediction using variables within a descriptive category. The table lists the performance for each potential model obtained using variables in a 'Variable Category' as the only predictors.

Variable Category	Mean AUC	Std AUC	Variable Category	Mean AUC	Std AUC
Oocyte & Embryo[a]	0.6437	0.0067	Serologic Hormone Values[e]	0.6027	0.0076
Age[b]	0.6018	0.0077	Lifestyle[f]	0.5356	0.0036
Sperm[c]	0.5258	0.0079	Race[g]	0.5244	0.005
Diagnoses[d]	0.5216	0.0092	BMI[h]	0.5016	0.0055

[a]Oocyte & Embryo: Oocytes retrieved, number of cryopreserved embryos, number of transferred embryos. [b]Age: age <35, age 35-37, age 38-40, age 41-42, age 42+. [c]Sperm: Sperm volume, concentration, motility, and progression. [d]Diagnoses: Female infertility, male infertility, unexplained infertility, tubal factor infertility, endometriosis, and ovulatory dysfunction. [e]Serologic Hormone Values: Day 3 ovarian test values: estradiol (E2), luteinizing hormone (LH), follicle stimulating hormone (FSH), endometrial thickness, antral follicle count (AFC), max estradiol, last anti mullarian hormone (AMH) value. [f]Lifestyle: Smoke (current, yes/no), alcohol use (current, yes/no), exercise (current, yes/no). [g]Race: White/Caucasian, Asian, Hispanic/Latina, Black/African American, unknown. [g]BMI: <18.5, 18.5-24.9, 25.5-29.9, 30.0-34.9, 35-39.9, 40+.

Supplementary Table S3. Race variable categories ranked by absolute value.

Rank	Variables	Coef	Coef 95% CI	Y1 mean	Y0 mean	p-value	Y-corr	Odds Ratio	Odds Ratio 95% CI
9	Race White/Caucasian	0.085	[0.06, 0.11]	0.22	0.18	<0.001	0.06	1.088	[1.057, 1.121]
18	Race Black/African American	-0.036	[-0.06, -0.01]	0.01	0.01	1.32E-02	-0.02	0.965	[0.938, 0.993]
31	Asian	-0.017	[-0.04, 0.01]	0.04	0.04	9.42E-01	0.00	0.983	[0.956, 1.011]
38	Hispanic/Latina	0.003	[-0.02, 0.03]	0.02	0.02	9.25E-01	0.00	1.003	[0.976, 1.031]

Supplementary Table S4. Epidemiological variables ranked by absolute value.

Rank	Variables	Coef	Coef 95% CI	Y1 mean	Y10 mean	P-value	Y-corr	Odds Ratio	Odds Ratio 95% CI
10	Currently Exercise	0.072	[0.04, 0.10]	0.31	0.25	<0.001	0.07	1.075	[1.042, 1.108]
20	Alcohol consumption	0.009	[-0.02, 0.04]	0.39	0.34	<0.001	0.05	1.009	[0.979, 1.040]

Supplementary Table S5. List of variables with missing values and breakdown of percentages by each variable.

Variables with missing values	Percentage of missing values
Day 3 antral follicle count (AFC)	84%
Race	73%
Day 3 endometrial thickness	67%
Day 3 luteinizing hormone (LH)	64%
Last anti mullarian hormone (AMH) Value	64%
Currently exercise	58%
Sperm progression	45%
Drink alcohol	44%
Diagnosis	42%
Day 3 estradiol (E2)	35%
BMI	22%
Day 3 follicle stimulating hormone (FSH)	22%
Sperm concentration	9%
Sperm volume	8%
Max estradiol (E2)	8%
Sperm motility	5%
